# Quality of Service and Cost of Managing an Episode of Major Depressive Disorder: A Cohort Study in Iran

**DOI:** 10.34172/aim.34141

**Published:** 2026-01-01

**Authors:** Masoumeh Amin-Esmaeili, Shahab Baheshmat, Afarin Rahimi-Movaghar, Seyed Mehdi Samimi Ardestani, Roya Samadi, Ahmad Hajebi, Yosef Farzy, Ebrahim Moghimi-Sarani, Shahab Khatibzadeh, Saeid Shahraz

**Affiliations:** ^1^Mental Health Department, Johns Hopkins Bloomberg School of Public Health, Baltimore, MD, USA; ^2^Department of Neuroscience and Addiction Studies, School of Advanced Technologies in Medicine (SATiM), Tehran University of Medical Sciences, Tehran, Iran; ^3^Iranian National Center for Addiction Studies (INCAS), Tehran University of Medical Sciences, Tehran, Iran; ^4^Departments of Psychiatry, Imam Hossein Hospital, Shahid Beheshti University of Medical Sciences, Tehran, Iran; ^5^Research Center for Psychiatry and Behavioral Sciences, Shiraz University of Medical Sciences, Shiraz, Iran; ^6^Research Center for Addiction and Risky Behaviors (ReCARB), Psychiatric Department, Iran University of Medical Sciences, Tehran, Iran; ^7^Non-Communicable Diseases Research Center, Endocrinology and Metabolism Population Sciences Institute, Tehran University of Medical Sciences, Tehran, Iran; ^8^Research Center for Psychiatry and Behavior Science, Shiraz University of Medical Sciences, Shiraz, Iran; ^9^Heller School of Social Policy and Management, Brandeis University, Waltham, Massachusetts, USA; ^10^Tufts Medical Center, Institute for Clinical Research and Health Policy Studies, Boston, MA, United States

**Keywords:** Affective disorders, Depression, Economic evaluation, Health service

## Abstract

**Background:**

Major depressive disorder (MDD), a major cause of the burden of diseases, is associated with a considerable rate of inadequate treatment and high costs. We assessed the pattern of service use, quality, and costs of healthcare for MDD in Iran.

**Methods:**

We assessed a national sample of 265 patients in the acute phase of MDD recruited from outpatient/inpatient settings at baseline and in three follow-up points of one, three, and six months. The pattern of service use and selected quality indicators were assessed, and the costs of care for an episode of MDD were estimated using the bottom-up approach.

**Results:**

The subjects were primarily female (73.6%), with a mean age of 43.3 years (±13.8). Of 173 respondents at the end of the study, 65.3% were on treatment for at least six months. Regarding quality indicators, the majority of the patients (97.7%) were prescribed antidepressants. However, only 71.2% of the patients initiated their antidepressants following prescription at the initial visit. At the end of the study, 40.1% were in remission and 58.1% had at least a 50% improvement in depressive symptoms. However, no standardized process or outcome measures were documented on the patients’ medical records. The average out-of-pocket and total costs for an episode of MDD per patient were estimated at Int$ 1331.4 and 2107.4, respectively.

**Conclusion:**

We recommend establishing an infrastructure for monitoring the clinical evaluation and treatment, and proper documentation based on standard quality of care, especially for ambulatory clinical practice.

## Introduction

 Major Depressive Disorder (MDD) is a common psychiatric disorder with a global estimate of 3.8% in 2019.^1^ It is associated with significant functional impairment and is considered a major cause of non-fatal burden.^2^ According to Global Burden of Diseases in 2019, MDD ranks second among 25 leading disorders with a high number of years lived with disability.^3^

 Despite efforts made over the past decades to improve mental health literacy and treatment-seeking behaviors through public mental health awareness along with training of healthcare professionals for early detection and treatment of MDD,^4,5^ a vast majority of those who suffer from MDD have an unmet need for treatment or barely receive a minimally adequate treatment.^6^ A large international study found that less than 17% of individuals with MDD in the past 12 months received minimally adequate treatment with a significant disparity between countries based on their income level.^7^ In high-income nations, 1 in 5 receives adequate treatment compared to 1 in 27 in low or lower-middle-income countries.^7^

 Iran is a lower-middle-income country^8^ with a relatively high prevalence of MDD.^9^ The Iranian Mental Health Survey (IranMHS) found that 12.7% of adults aged 15‒64 years had MDD and about 40% of them utilized health services,^10^ albeit it was an inadequate treatment in almost half of those who received any health services.^10^ According to this study, the stigma associated with mental disorders, financial barriers, and low insurance coverage play a significant deterrent role in the health-seeking behaviors of individuals with MDD in Iran and impact healthcare utilization. Despite the expansion of mental health programs in the urban primary healthcare system during the last decade, economic sanctions and the inflation rate might hinder access to medicines and non-public healthcare services and widen health inequalities.^11,12^

 MDD imposes significant economic burdens due to high healthcare utilization, work absence, and productivity loss.^13-16^ A recent meta-analysis of cost-of-illness studies found that depression causes 158% and 128% extra direct and indirect costs, respectively, in depressed versus non-depressed adults.^13^ However, the majority of the studies included in the meta-analysis were from high-income countries, primarily the US and European countries, where there is high-quality claims data or Electronic Medical Records and patients have a high insurance coverage.

 Very few studies have estimated the cost of depression in a low- or middle-income country.^13,16,17^ The existing literature on these countries primarily comprises retrospective studies, limiting the study to particular types of cost (e.g. out-of-pocket costs, direct costs), targeting certain types of patients (e.g. perinatal depression, depression in the elderly, or children), or conducting the study on a non-representative sample. There is much to be known about the costs of MDD in low- or middle-income countries.

 The current study is part of a national-based longitudinal study of seven common medical conditions in Iran including myocardial infarction, congestive obstructive pulmonary disease,^18^ congestive heart failure, diabetes mellitus,^19^ stroke, end-stage renal failure, and major depressive disorder. The pattern of service use, quality, and costs of healthcare for MDD are described in this report. In the current study, we identified the pattern of service use and investigated the quality of the services through a longitudinal study design on a representative sample of the adult population diagnosed with MDD. Additionally, we estimated the cost of care for an episode of MDD, both direct and indirect, and catastrophic health expenditure using a bottom-up approach.

## Materials and Methods

###  Design & Sampling Method

 A representative national sample was recruited using hierarchical and model-based cluster sampling with further details available elsewhere.^20,21^ In summary, the decision tree machine learning method was applied to initially divide the country into eight clusters based on the available data on health care structure, service utilization, and outcome for the selected conditions. The province closest to the center of each super-cluster was then selected. For each selected province (i.e. Tehran, West Azerbaijan, South Khorasan, Fars, Khuzestan, Qazvin, Lorestan, and Guilan), psychiatrists practicing in public or private clinics in the provincial capital were invited to participate in the study. Consistent with the IQCAMP protocol, the study aimed to recruit approximately 300 patients per condition to balance feasibility with intensive clinical, utilization, cost, and quality data collected for each participant. Psychiatrists who agreed to participate provided patient information and medical records after obtaining the necessary permissions from patients. Thirteen psychiatric nurses, psychologists, or medical students working in the selected clinics or hospitals conducted the interviews. They received comprehensive training on reassessing and obtaining written informed consent, administering baseline and follow-up measurements, and reviewing the patients’ medical records.

###  Participants

 Patients were eligible to participate in the study if they 1) were 18 years of age or older; 2) had a diagnosis of MDD according to the Diagnostic and Statistical Manual of Mental Disorders fifth edition (DSM-5)^22^ by a psychiatrist; 3) were in the acute phase of the disorder, 4) were willing to participate in the study; 5) provided the address and phone number for follow-up contact, and 6) resided in the city where the treatment center was located. The acute phase was defined as symptom onset or worsening within three months, evidenced by treatment initiation or change. Patients were excluded if they had a history of bipolar disorder, or were diagnosed with dysthymia, premenstrual dysphoric disorder, grief reaction (depression within two months following the death of loved ones), adjustment disorder with depressed mood, and depression due to medical illness or substance use.

###  Measurements

 The included patients were initially assessed at baseline and re-interviewed in three follow-up points, one, three, and six months after enrollment at their clinical encounter. A computer-assisted personal interview was developed using the Java programming language and MS-SQL for database maintenance with the capability of both online and offline data collection. Detailed information about the design of the program is published elsewhere.^21^ Information about demographics, severity of depression, functional impairment, risk of suicide, service utilization, and treatment adherence was collected in each interview using standard instruments including Patient Health Questionnaire-9 (PHQ-9)—a screening tool to quantify depression symptoms and monitor severity,^23^ suicide assessment questionnaire—to identify the suicidal thoughts, plan and attempts over the study period,^24^ the Sheehan Disability Scale,^25^ and a detailed questionnaire on service utilization, adherence to treatment, and cost assessment adapted from the related tools of the Iranian Mental Health Survey (IranMHS).^26^ The Sheehan Disability Scale (SDS) assesses disability status in three domains of activities (household and family life, work & school responsibilities, and social life) using 10-point visual and numeric descriptive anchors.^25^ The Persian version of SDS was well-validated^25^ and it was already used for monitoring the treatment effect over the course of psychiatric disorders.^27-29^ The total score of the SDS was calculated (ranging 0-30) with a higher score indicating greater impairment. The functional abilities were assessed using two indicators 1) functional response (i.e. total score ≤ 12) and functional remission (i.e. total score ≤ 6).^28^ Using our assets questionnaire, we employed principal component analysis to calculate the wealth index which was categorized into five groups “very low” to “very high wealth” index.

 To quantify the quality of care, we adopted a number of quality indicators provided by the National Quality Forum (NQF) for depression,^30^ as well as the US Center for Quality Assessment and Improvement in Mental Health (CQAIMH).^31^ The indicators were modified according to the structure of health care provision in Iran through a panel discussion with the experts in the field of treatment of mood disorders and familiar with the practice guidelines for the treatment of patients with MDD.^32^ The list of quality indicators along with the definition and method of assessment are presented in Table 1.

**Table 1:** Quality indicators for treatment of Major Depressive Disorders (MDD)

**QI-ID**	**Quality Indicators**	**Definition**	**Assessment Method**
QI-1	Assessment for manic or hypomanic episodes	The patient was assessed for current and/or prior symptoms of a manic or hypomanic episode at the initial visit	Based on the provider’s self-report and review patient’s record
QI-2	Assessment for alcohol or substance use	The patient was screened for current alcohol or substance use at the initial visit	Based on the provider’s self-report and review patient’s record
QI-3	Assessment of suicide risk	The patient was assessed for current and/or prior suicidal thoughts, plans, and/ or attempts at initial and follow-up visits	Based on the patient’s self-report, and review patient’s record
QI-4	Initiation of antidepressant medication	An antidepressant was prescribed by the provider and the patient filled out the prescribed medication	Based on the patient’s self-report and reviewing the patient’s record
QI-5	Adherence	The patient used the prescribed medications almost regularly, or completely regular	Based on the patient’s self-report at each follow-up assessment point
QI-6	Remained on antidepressant treatment for at least 3 months	The patient used the prescribed medication irregularly/ almost regularly, or completely regular for at least 3 months	Based on the patient’s self-report
QI-7	Remained on treatment for at least 6 months	The patient used the prescribed medication irregularly/ almost regularly, or completely regular for at least 6 months	Based on the patient’s self-report
QI-8	In remission 6 months after initiation of treatment	The patient had a PHQ-9 score < 5, six months after initiation of treatment	Based on the patient’s self-report to PHQ-9 at baseline and the last follow-up assessments
QI-9	Had at least 50% improvement after initiation of treatment relative to baseline score	Patients had at least 50% improvement in PHQ-9 score, 6 months after starting treatment-comparing to baseline score	Based on the patient’s self-report to PHQ-9 at baseline and the last follow-up assessments
QI-10	Functional response	The patient had a SDS total score < 12, six months after initiation of treatment	Based on the patient’s self-report to SDS at the last follow-up assessments
QI-11	Functional remission	The patient had a SDS total score < 6, six months after initiation of treatment	Based on the patient’s self-report to SDS at the last follow-up assessments

PHQ-9, Patient Health Questionnaire-9; QI-ID, Quality Indicator Identification Number; SDS, Sheehan Disability Scale.

 To measure the cost of an episode of care for MDD, a bottom-up approach was used.^33^ In the first step for assessment of the direct cost of an MDD episode, the amount of health care services was determined by asking the participants about their service use for the MDD. The use of outpatient and inpatient health care services was assessed which includes hospital admission, any outpatient service such as outpatient visits by a psychiatrist or other physicians, a psychologist or a psychotherapist and other health care workers, receiving outpatient electro-convulsive therapy (ECT), medications, laboratory, and imaging services. In the next step, the out-of-pocket cost of each service was evaluated based on patients’ self-report using a modified questionnaire adopted from the IranMHS cost assessment instrument^34^ as well as reviewing the patients’ medical bills. We also asked whether the services were covered by insurance. In addition to direct health care costs, there were questions regarding the direct non-health cost which includes any cost for the patient or the caregivers’ transportation, accommodation, the cost of hiring a nurse or caregiver, and the patient’s time spent if they were related to the received health care services. The indirect cost of MDD was also measured by asking about the cost of caregivers’ time spent, patients’ and caregivers’ cost of absenteeism, loss of productivity, and family leisure time. At the baseline, patients were asked about the service they received for MDD since the initiation of treatment for the current MDD episode. The same questions were asked at the follow-up assessments along with the questions regarding the cost of services patients received during the period between the previous assessment and current assessment (past month at the 1^st^ follow-up, and past two and three months in the 2^nd^ and 3^rd^ follow-ups, respectively). Cost outcomes were summarized descriptively using means, standard deviations, medians, and interquartile ranges.

 We also assessed whether the patient experienced catastrophic health expenditure during the MDD episode which exposed them to financial hardship by asking whether they 1) lost their savings due to direct payment for the health services, 2) had to get a loan to pay the cost related to MDD.

###  Analyses

 We described the baseline sociodemographic characteristics of the total sample and assessed whether they differed between patients who completed all follow-up assessments and those lost to follow-up. Odds ratios (ORs) and their 95% confidence intervals were estimated using logistic regression models with follow-up completion as the binary outcome. A two-sided *P*-value < 0.05 was considered statistically significant. The quality of MDD care was presented by the percentage of patients who fulfilled each quality measure in the total sample and stratified by sex. We obtained *P*-values using chi-square tests to compare males and females.

 The cost of care for an episode of MDD was calculated by the sum of direct health and non-healthcare costs, as well as the indirect costs, limiting the sample to those who attended all the follow-up assessments (n = 164). The cost was calculated for an episode of care which was considered a period between 6 to 9 months. It is the recommended duration of treatment for an acute major depressive episode.^32,35^ Based on the general health insurance benefit package provided by public and private Iranian insurance companies, the unit cost was roughly corrected by considering 70% and 90% insurance coverage for outpatient and inpatient services, respectively.^36-38^ For those patients whose treatment had been started before the initiation of the study, we collected data on all services received during the period between treatment initiation and the baseline assessment point, but we had a missing value for the cost of services they received in this period. Using the information we collected in the follow-up assessments, we calculated the average unit cost of each service (e.g. the unit cost of an outpatient visit), accounting for insurance coverage. Then, we multiplied the estimated unit cost by the number of given services received during the period between treatment initiation and the baseline assessment point. We presented the average cost per service and total cost among those who used the services for each category of direct and indirect costs and their subcategories.

 The indirect cost was calculated by estimation of daily income based on the minimum daily wage of Iranian employers in the year 2018, which was modified for marital status and number of children, multiplied by workdays missed due to MDD. Additionally, we determined what percentage of the total cost was spent on each category of the cost. We also provided the proportion of patients who had catastrophic health expenditures.

 All the estimated costs in Iranian Rials were converted to Purchasing Power Parity (PPP) (each Int$ PPP was equal to 16770.2 IRR in 2018).^39^ Using the national estimate of the adult population who was diagnosed with MDD in the past 12 months (12.7% of the total adult population) and those who received minimally adequate treatment,^10^ we additionally estimated the economic burden of MDD for those who were treated in the country.

###  Ethical Considerations

 For ethical considerations, the purpose, outcomes, and adverse effects of the study were explained to patients, and written informed consent was obtained. The treatment was not affected if the patients were reluctant to participate in the study. In addition, patients were assured of confidentiality and anonymity of the participants’ information. The ethics committee of the Iran National Institute for Medical Research Development (NIMAD) approved the study protocol (Ethics code: IR.NIMAD.REC.1395.003).

## Results

###  Description of the Sample

 A total of 265 patients with major depressive disorders completed the baseline assessment (Appendix 1). The distribution of the sample recruited from eight provinces ranged from 6.5% in Qazvin to 24.5% in Guilan. The majority of the sample were female (73.6%), with a mean age of 43.3 ( ± 13.8) ranging from 18 to 94 years and a median age of 42 years. Of the total sample, 227 patients (85.7%) were interviewed at least once during the follow-up period, and 164 patients (61.9%) participated in all the follow-up assessments. There was no significant sociodemographic difference between patients who were lost to follow-up and those who participated in all the assessments except for the wealth index; compared to patients with a very low wealth index, those with a very high wealth index were more likely to attend all follow-up visits (Odd Ratio = 3.4, 95% Confidence Interval: 1.04, 10.8; *P* = 0.042). The average days of follow-up of those 227 patients were 153.2 ( ± 44.4) days. Of the total 262 patients, 224 patients (84.5%) participated in the first follow-up interview, 195 (73.6%) in the second, and 173 (65.3%) in the third interview.

###  Healthcare Quality 

 The quality of healthcare services is presented in Table 2. Suicide risk assessment at the initial visit, which is a fundamental screening procedure among MDD patients, was either missed or not documented in about one-fifth of the cases. The percentage of suicide screening further decreased in the next follow-up assessments. The majority of the patients (97.7%) were prescribed antidepressants and filled their prescriptions at some point during their MDD episode; however, only 71.2% of the patients initiated their antidepressants following prescription at the initial visit. Treatment adherence diminished during the episode as it was 92.9%, 71.3%, and 69.4% at the first, second, and third follow-ups respectively. Of 173 respondents at the end of the study, 65.3% were on treatment for at least 6 months. According to the PHQ-score, 40.1% were in remission at the end of the study and 58.1% had at least 50% improvement in depressive symptoms. Functional response and functional remission were observed in 73.3% and 39.5% of the cases at the end of follow-up, respectively. No significant differences were observed between males and females in most of the indicators with a few exceptions. Males were less likely to be initiated with an antidepressant (*P* = 0.001), and to have a functional response (*P* = 0.044) or functional remission compared to females (*P* value = 0.023). No significant differences were observed between males and females across most quality indicators. For antidepressant initiation, the proportion appeared somewhat lower among males (92.8%, 95% CI: 83.7–97.0) than females (99.5%, 95% CI: 96.4–99.9; *P* = 0.001). At the 3-month follow-up, males also showed lower proportions of functional response (65.9%, 95% CI: 50.2–78.7) compared with females (80.9%, 95% CI: 73.2–86.8; *P* = 0.044), and lower proportions of functional remission (24.4%, 95% CI: 13.6–39.8) compared with females (44.3%, 95% CI: 40.0–52.9; *P* = 0.023).

**Table 2:** Quality indicators of care services for MDD

**QI-ID**	**Indicator**	**Assessment Point**	**Respondents ** **N**	**Total**		**Male**		**Female**	
				***n***	**%**	***n***	**%**	***n***	**%**
QI-1	Assessment of Mania/hypomania	Initial visit	260	244	93.9 (90.2-96.2)	66	95.7 (87.3-98.6)	178	93.2 (88.6-96.0)
QI-2	Assessment of substance use	Initial visit	261	233	89.3 (84.9-92.5)	65	94.2 (85.5-97.8)	168	87.5 (82.0-91.5)
QI-3	Suicide risk assessment	Initial visit	259	212	81.9 (76.7-86.1)	56	82.4 (71.3-89.7)	156	81.7 (75.5-86.6)
QI-3	Suicide risk assessment	1^st^ FU	217	180	83.0 (77.3-87.4)	37	80.4 (66.4-89.5)	143	83.6 (77.2-88.5)
QI-3	Suicide risk assessment	2^nd^ FU	180	130	72.2 (65.2-78.3)	27	65.9 (50.2-78.7)	103	74.1 (66.1-80.7)
QI-3	Suicide risk assessment	3^rd^ FU	169	105	62.1 (54.5-69.2)	24	58.5 (43.0-72.5)	81	62.3 (54.5-71.2)
QI-4	Antidepressant initiation	Initial or FU visits	262	256	97.7 (95.0-99.0)	64	92.8 (83.7-97.0)	192	99.5 (96.4-99.9)
QI-4	Antidepressant initiation	Initial visit	262	189	72.7 (66.9-77.7)	50	74.6 (62.9-83.6)	139	72.0 (65.2-77.9)
QI-4	Adherence	1st FU	224	208	92.9 (88.6-95.6)	44	93.6 (81.9-97.9)	164	92.6 (87.7-95.7)
QI-5	Adherence	2^nd^ FU	195	139	71.3 (64.5-77.2)	31	68.9 (54.0-80.7)	108	72.0 (64.2-78.6)
QI-5	Adherence	3^rd^ FU	173	120	69.4 (62.0-75.8)	32	76.2 (61.0-86.7)	88	67.2 (58.6-74.7)
QI-6	On treatment-3 mths	2^nd^ FU	194	180	92.8 (88.1-95.7)	39	86.7 (73.2-93.9)	141	94.6 (90.0-97.3)
QI-7	On treatment-6 mths	3^rd^ FU	171	113	66.1 (58.6-72.8)	28	66.7 (51.2-79.2)	85	65.9 (57.2-73.6)
QI-8	On remission	3^rd^ FU	172	69	40.1 (33.0-47.7)	19	46.3 (31.8-61.6)	50	38.2 (30.2-46.8)
QI-9	50% improvement	3^rd^ FU	172	100	58.1 (50.6-65.3)	23	56.1 (40.7-70.4)	77	58.8 (50.1-66.9)
QI-10	Functional response	3^rd^ FU	172	133	73.3 (70.4-83.0)	27	65.9 (50.2-78.7)	106	80.9 (73.2-86.8)
QI-11	Functional remission	3^rd^ FU	172	68	39.5 (32.5-47.1)	10	24.4 (13.6-39.8)	58	44.3 (40.0-52.9)

Mths, Months, FU, Follow-up assessment.

###  Service Utilization and Cost

 Individuals who did not participate in any of the three follow-ups were excluded from the cost analyses (n = 164). The patients received treatment and care for an average of 7.0 ( ± 1.3) months. The average outpatient and inpatient visits in an episode of MDD were about 5 and 0.2, respectively (Table 3). Patients were rarely examined by some sort of diagnostic services (e.g. imaging, lab tests). All of the 164 respondents were prescribed antidepressants.

**Table 3:** Health service utilization of patients during an episode of MDD

	**No. of Respondents Who Received Services**	**No. of Services Received**	**Services used by Total Respondents (** ***n*** **=164)**		
			**Mean (SD)**	**Median**	**Range**
Total outpatient visits					
Total	164	874	5.3 (2.6)	5	1-16
Male	40	242	6.1 (3.4)	5	2-16
Female	124	632	5.1 (2.3)	5	1-16
Hospital admission					
Total	20	24	0.2 (0.4)	0	0-3
Male	6	9	0.2 (0.6)	0	0-3
Female	14	15	0.1 (0.4)	0	0-2
Total medication visits					
Total	164	734	4.5 (2.2)	4	1-13
Male	40	202	5.1 (2.9)	5	1-13
Female	124	532	4.3 (1.8) 4	4	1-9
Laboratory services					
Total	39	43	0.3 (0.5)	0	0-2
Male	8	9	0.2 (0.5)	0	0-2
Female	31	34	0.3 (0.5)	0	0-2
Imaging services					
Total	6	6	0.04 (0.2)	0	1
Male	2	2	0.05 (0.2)	0	1
Female	4	4	0.03 (0.2)	0	1

 The average out-of-pocket and total cost for an episode of MDD per patient was estimated at Int$ 1331.4 and Int$ 2107.4 Purchasing Power Parity (PPP) (Table 4). The direct health cost was the largest part of the total cost (51.1%) which primarily included the cost of medications (22.7%), inpatient services (14.4%), and outpatient visits (9.6%). The indirect cost of the MDD episode was also quite substantial (33.2%). Of 164 respondents, 78 (47.6%) reported having catastrophic payment due to an MDD episode mainly due to spending the household savings to pay the medical bills.

**Table 4:** Service utilization, out-of-pocket, and total cost of an episode of Major Depressive Disorder (MDD) (N = 164)

	**No. Received Services**	**Out-of-pocket Cost per Patient per Episode (Int$-PPP)**					**Total Cost per Patient per Episode (Int$-PPP)**				
		**Those Received Services**		**Total Sample **			**Those Received Services**		**Total sample **		
		**Mean (SD)**	**Median (IQR)**	**Mean (SD)**	**Median (IQR)**	**% of the total cost**	**Mean ** **(SD)**	**Median (IQR)**	**Mean ** **(SD)**	**Median ** **(IQR)**	**% of the total cost**
Total cost (Int$)	164	1331.4 (1951.4)	635.6 (927.6)	1331.5 (1951.4)	635.6 (927.6)	100	2107.6 (2514.4)	1210.4 (1,722.4)	2107.6 (2514.4)	1210.4 (1,722.4)	100
Direct health cost	164	300.8 (329.4)	188.8 (254.4)	300.8 (329.4)	188.8 (254.4)	22.6	1077.0 (1426.5)	592.4 (1013.9)	1077.0 (1426.5)	592.4 (1013.9)	51.1
Any Therapeutic Services	164	270.7 (306.9)	164.6 (195.8)	270.7 (306.9)	164.6 (195.8)	20.3	984.7 (1342.6)	555.2 (834.2)	984.7 (1342.6)	555.2 (834.2)	46.7
Inpatient services	20	354.2 (500.5)	221.5 (236.9)	43.2 (206.7)	0 (0)	3.2	249.0.4 (2297.9)	2138.1 (2118.8)	303.7 (1133.0)	0 (0)	14.4
Outpatient services	164	76.3 (112.6)	29.6 (67.8)	76.3 (112.6)	29.6 (67.8)	5.7	201.8 (278.0)	83.9 (179.4)	201.8 (278.0)	83.9 (179.4)	9.6
Medications	164	151.2 (164.6)	108.1 (98.5)	151.2 (164.6)	108.1 (98.5)	11.4	479.2 (472.4)	326.4 (367.4)	479.2 (472.4)	326.4 (367.4)	22.7
Diagnostic Services	40	123.6 (82.3)	89.4 (150.3)	30.2 (66.7)	0 (0)	2.3	378.2 (254.6)	253.4 (461.5)	92.2 (205.1)	0 (0)	4.4
Laboratory	39	114.0 (73.3)	73.8 (152.9)	27.1 (60.2)	0 (0)	2.0	345.1 (222.9)	223.8 (472.6)	82.1 (182.4)	0 (0)	3.9
Imaging	6	83.5 (0)	83.5 (0)	3.1 (15.7)	0 (0)	0.2	278.2 (0)	278.2 (0)	10.2 (52.4)	0 (0)	0.5
Direct non-health cost	164	330.3 (415.2)	302.2 (162.2)	330.3 (415.2)	302.2 (162.2)	24.8	330.3 (415.2)	302.2 (162.2)	330.3 (415.2)	302.2 (162.2)	15.7
Transportation/ travel	160	72.3 (98.2)	47.6 (32.4)	70.5 (97.7)	47.6 (32.2)	5.3	72.3 (98.2)	47.6 (32.4)	70.5 (97.7)	47.6 (32.2)	3.3
Accommodations & food cost	47	50.1 (69.9)	28.0 (45.4)	14.4 (43.5)	0 (12.9)	1.1	50.1 (69.9)	28.0 (45.4)	14.4 (43.5)	0 (12.9)	0.7
Patients’ time spent	164	244.2 (370.9)	227.0 (191.6)	244.2 (370.9)	227.0 (191.6)	18.3	244.2 (370.9)	227.0 (191.6)	244.2 (370.9)	227.0 (191.6)	11.6
Hiring nurse/caregiver	1	195.0 (NA)	195.0 (0)	1.2 (15.2)	0 (0)	0.09	195.0 (NA)	195.0	1.2 (15.2)	0 (0)	0.06
Indirect cost	108	1063.4 (1926.9)	413.4 (809.1)	700.3 (1641.1)	15.2 (613.5)	52.6	1063.4 (1926.9)	413.4 (809.1)	700.3 (1641.1)	15.2 (613.5)	33.2
Caregivers’ time spent	87	326.0 (659.0)	115.3 (273.9)	116.3 (378.7)	0 (59.3)	8.7	326.0 (659.0)	115.3 (273.9)	116.3 (378.7)	0 (59.3)	5.5
Patients’ Absenteeism	20	463.9 (799.0)	166.8 (256.8)	56.6 (312.4)	0 (0)	4.3	463.9 (799.0)	166.8 (256.8)	56.6 (312.4)	0 (0)	2.7
Caregivers’ Absenteeism	41	237.4 (735.1)	82.3 (74.4)	59.4 (378.4)	0 (20.3)	4.5	237.4 (735.1)	82.3 (74.4)	59.4 (378.4)	0 (20.3)	2.8
Patients’ productivity loss	15	1527.6 (2473.2)	686.8 (1,053.3)	139.7 (848.8)	0 (0)	10.5	1527.6 (2473.2)	686.8 (1,053.3)	139.7 (848.8)	0 (0)	6.6
Caregivers’ productivity loss	61	563.6 (825.5)	258.0 (505.9)	209.6 (570.5)	0 (154.7)	15.7	563.6 (825.5)	258.0 (505.9)	209.6 (570.5)	0 (154.7)	9.9
Family leisure time spent	28	695.7 (1203.4)	157.0 (752.6)	118.8 (555.7)	0 (0)	8.9	695.7 (1203.4)	157.0 (752.6)	118.8 (555.7)	0 (0)	5.6

NA, Not applicable; SD, Standard Deviation; IQR, Interquartile range; PPP, Purchasing Power Parity. *The denominator is the total sample who remained in the study until the end of follow-up.

 Given the national prevalence of MDD (12.7% of the population aged 15‒64 years),^10^ the economic burden of an episode of MDD is estimated to be about Int$ 14.7 billion PPP. The estimates would be about Int$ 6.1 billion PPP if we only took into account the proportion of adult patients who received any health services (41.4% of adult patients = 2,901,597 people).

## Discussion

 In the current study, we determined the pattern of service utilization, quality of care, and cost of an episode of MDD in a real-world situation through a longitudinal study on a representative sample of Iranian patients with an acute episode of MDD who initiated a treatment plan for the MDD. The key finding of the study is the estimated average cost for an episode of MDD per patient which was Int$ 2107.6, comprising 14% of the gross domestic product (GDP) per capita in Iran in the year 2018.^40^ It translates to an economic burden of over Int$ 6 billion for the estimated number of people with the diagnosis of MDD who receive any health care services and nearly Int$ 15 billion if we take into account all the patients in need of health care services in the country.

 The cost estimate of the current study is consistent with prior studies in Iran^41^ and other countries^16^ in which the average annual direct cost of MDD ranged between $1000 to $2500. In a study conducted in the year 2020 in a province located in southern Iran, Keshavarz et al. reported an annual direct cost of around Int$2200 PPP per patient.^41^ The difference with our study is partly due to the timeframe of the cost calculation which was the cost for an episode of MDD in our study (an average of 7 months) and the annual cost in the study by Keshavarz et al. Moreover, the time of study was two years later compared to our study when the country was experiencing a sharp increase in inflation rates escalated by newly imposed economic sanctions which might be a contributing factor to the overall higher cost of illness in the study by Keshavarz et al. Furthermore, the sample was limited to one geographical area and recruited more severe patients who had a history of hospital admission while the current study benefited from a nationally representative sample of patients with a history of either inpatient or outpatient service utilization for MDD.

 As expected, our estimate was lower than the studies conducted in high-income countries such as USA and Canada with over USD 6000^42^ and CAD 10,000^43^ per treated patient. Healthcare services, in general, and medicines in particular are cheaper in Iran than in some other countries partly because of substantially high subsidies allocated to medicines by the Iranian government.^44^ Moreover, the government controls the prices by setting price ceilings. Examples of prices in Iran are: an outpatient visit by psychiatrist costs less than $10, the tariff of a brain MRI without contrast is less than $100, the cost for 20 mg oral citalopram tablets for a supply of 30 tablets is around $1 in Iran, while the corresponding figures are around $500, $1000‒$12000 and $35 in the US, respectively, depending on location, the facility, complexity of the problem, and whether the patient is cash payer or insured. Although the health care cost seems cheap, it is not affordable for an Iranian household whose average annual income is nearly Int$ 2600.^45^ This comparison also supports the other findings of our study indicating that the financial hardship of MDD through catastrophic payment affected approximately half of the patients. Our study showed that the out-of-pocket direct cost of an episode of MDD is over Int$ 600, which is about a quarter of the annual income of an urban Iranian household. Therefore, some of the patients had to spend their savings or get loans to be able to pay their medical bills.

 The findings regarding the pattern of service utilization indicate that the MDD patients attended about five outpatient visits on average during an episode of depression which accounts for less than one visit per month for an average of 7 months of follow-up. A small proportion of the patients were hospitalized, and they rarely underwent a laboratory test or diagnostic imaging. There is no standard frequency for outpatient visits that applies to all patients with MDD in the acute phase. According to the practice guidelines of American Psychiatric Association, the number of follow-up visits varies from once a week to multiple times a week depending on severity of illness, type of treatment (psychotherapy, medication only, or a combination of both), whether it is within the first three months of episode or in continuation or maintenance phase, availability of social and family support, presence of comorbid medical or other psychiatric disorders which require closer monitoring of the clinical improvement, potential medications’ side-effects and the drug interactions.^46^ However, the frequency of visits is generally less than optimal in community settings^7,47^ than in controlled settings like randomized clinical trials which is the main source of evidence for developing practice guidelines. In a population-based study in Montreal, fewer than 50% of the newly diagnosed MDD patients had an optimal frequency of physician contacts defined as having at least three follow-up visits during the first three months of the MDD episode.^47^

 Consistent with the existing literature,^48-53^ a significant portion of patients at follow-up exhibited positive outcomes: regular antidepressant use (around 70%), remission (40% with PHQ-9 score < 5), 50% or greater improvement in the PHQ-9 score (58%), and acceptable functional response (over 70%). Additionally, over 66% continued antidepressant treatment for at least six months. The existing literature supports the effectiveness of some of the quality measures in monitoring and properly quantifying and recording the treatment success including assessment of symptom severity by the PHQ-9 score, or functional improvement using a standardized scale such as SDS.^54-56^ However, we found no standardized process or outcome measures documented in the patients’ medical records, mainly because no clinical practice guideline has been developed or adopted from available standard guidelines for mental health professionals working in public or private healthcare facilities in the country. Furthermore, there is no infrastructure for recording administrative data except for hospital admissions. The providers are not required to systematically monitor the patients using self-reported scales. No prior study has examined the quality of care provided for MDD patients in Iran. Quality indicators were adapted through expert panel discussion, yet their real-world applicability in Iran remains uncertain. Further research is needed to validate quality indicators for MDD treatment in Iran’s healthcare setting.

## Limitations

 The study had several limitations to consider. First, nearly two-thirds of the participants were interviewed at all assessment points. However, those who persisted had no significant sociodemographic difference compared to those who were lost the follow-up, except for the wealth index. Those who continued the visits had a higher wealth index than those who left the study, justifying the financial factor contributing to drop-out. Second, we excluded the patients who had other comorbid psychiatric disorders, substance use disorders, or medical illnesses to be able to exclusively estimate the cost of an MDD episode; therefore, the cost might have been underestimated considering a high probability of comorbidity among patients diagnosed with MDD. However, the sociodemographic characteristics were consistent with the description of people with MDD in the community,^10^ confirming a representative sampling procedure. MDD patients in our study were primarily females, middle-aged, married, with an education level lower than a diploma. The only significant difference is that a higher proportion of our sample had a high or very high wealth index compared to the national estimate, suggesting that those who can afford the treatment are more likely to initiate their treatment plan. Third, although the sampling design involved clustering at the provincial and clinic levels, the analyses did not incorporate cluster-level adjustments. This choice reflects the exploratory aim of the study and its focus on individual-level associations; however, lack of cluster adjustment may have modestly affected variance estimates and should be considered when interpreting the findings. Lastly, the patients in the current study were recruited from psychiatric care settings and the findings might not be generalized to all the MDD patients in the country because according to the IranMHS, about 50% of patients refer to GPs for treatment of depression.^10^

## Conclusion

 Our study found that MDD is associated with a substantial increase in direct and indirect costs, leading to a considerable burden on both the health system and the individual. Quality indicators were used to monitor healthcare performance for MDD, revealing the need for improvement in treatment quality. Despite this, treatment resulted in improved clinical outcomes in most cases.

 To prevent the progression and recurrence of MDD and further increases in the cost, strengthening the mental health resources, increasing coverage of services, using multidisciplinary teams for psychosocial care, and patient education and engagement are needed. Cost-effective prevention and intervention methods are necessary to reduce out-of-pocket and total costs.

## Appendix

**Appendix 1:** Demographic and clinical characteristics of the sample at baseline

	**Baseline**		**1** ^st^ ** Follow-up**		**2** ^nd^ ** Follow-up**		**3** ^rd^ ** Follow-up**	
	**N**	**%**	**N**	**%**	**N**	**%**	**N**	**%**
Total respondents	265	100	224	84.5	195	73.6	173	65.3
Sex								
Male	70	26.4	47	21.0	45	23.1	42	24.3
Female	195	73.6	177	79.0	150	76.9	131	75.7
Age group								
< 30	40	15.1	32	14.3	25	12.8	23	13.3
30-49	136	51.3	118	52.7	101	51.8	90	52.0
50-64	63	23.8	51	22.8	48	24.6	41	23.7
> 65	18	6.8	15	6.7	14	7.2	13	7.5
Refused to answer/missing	8	3.0	8	3.6	7	3.6	6	3.5
Education								
Illiterate	37	14.0	34	15.2	31	15.9	26	15.0
Primary school	54	20.4	45	20.1	38	19.5	34	19.7
Middle/high school	61	23.0	50	22.3	45	23.1	36	20.8
High school diploma	62	23.4	52	23.2	42	21.5	40	23.1
University graduate	40	15.1	37	16.5	34	17.4	32	18.5
Post-graduate degree	8	3.0	6	2.7	5	2.6	5	2.9
Refused to answer/missing	3	1.1	0	0	0	0	0	0
Marital status								
Never married	44	16.6	37	16.5	29	14.9	26	15.0
Married	185	69.8	157	70.1	139	71.3	123	71.1
Divorced	13	4.9	11	4.9	9	4.6	9	5.2
Widowed	19	7.2	18	8.0	17	8.7	14	8.1
Refused to answer/missing	4	1.5	1	0.4	1	0.5	1	0.6
Wealth index								
Very low	20	7.6	11	4.9	9	4.6	10	5.8
Low	37	14.0	26	11.6	16	8.2	14	8.1
Intermediate	42	15.8	39	17.4	37	19.0	31	17.9
High	80	30.2	70	31.2	61	31.3	52	30.1
Very high	86	32.5	78	34.8	72	36.9	66	38.2
Insurance								
Basic	247	94.3	—	—	—	—	—	—
Complementary	48	18.3	—	—	—	—	—	—

## References

[1] Castaldelli-Maia JM, Bhugra D (2022). Analysis of global prevalence of mental and substance use disorders within countries: focus on sociodemographic characteristics and income levels. Int Rev Psychiatry.

[2] GBD 2016 Disease and Injury Incidence and Prevalence Collaborators. Global, regional, and national incidence, prevalence, and years lived with disability for 328 diseases and injuries for 195 countries, 1990-2016: a systematic analysis for the Global Burden of Disease Study 2016. Lancet. 2017;390(10100):1211–59. doi: 10.1016/s0140-6736(17)32154-2 PMC5605509 28919117

[3] GBD 2019 Mental Disorders Collaborators (2022). Global, regional, and national burden of 12 mental disorders in 204 countries and territories, 1990-2019: a systematic analysis for the Global Burden of Disease Study 2019. Lancet Psychiatry.

[4] Prince M, Patel V, Saxena S, Maj M, Maselko J, Phillips MR (2007). No health without mental health. The Lancet.

[5] Patel V, Saxena S, Lund C, Thornicroft G, Baingana F, Bolton P (2018). The Lancet Commission on global mental health and sustainable development. Lancet.

[6] Moitra M, Santomauro D, Collins PY, Vos T, Whiteford H, Saxena S (2022). The global gap in treatment coverage for major depressive disorder in 84 countries from 2000-2019: A systematic review and Bayesian meta-regression analysis. PLoS Med.

[7] Thornicroft G, Chatterji S, Evans-Lacko S, Gruber M, Sampson N, Aguilar-Gaxiola S (2017). Undertreatment of people with major depressive disorder in 21 countries. Br J Psychiatry.

[8] Hamadeh N, Van Rompaey C, Metreau E. New World Bank country classifications by income level: 2022-2023. The World Banl. https://blogs.worldbank.org/opendata/new-world-bank-country-classifications-income-level-2022-2023. Published 2022. Accessed July 7, 2023.

[9] Gharraee B, Zahedi Tajrishi K, Sheybani F, Tahmasbi N, Mirzaei M, Farahani H (2019). Prevalence of major depressive disorder in the general population of Iran: A systematic review and meta-analysis. Med J Islam Repub Iran.

[10] Amin-Esmaeili M, Shadloo B, Rahimi-Movaghar A, Samimi Ardestani SM, Hajebi A, Khatibzadeh S (2022). Major Depressive Disorder in Iran: Epidemiology, Health Care Provision, Utilization, and Challenges. Arch Iran Med.

[11] Aloosh M, Salavati A, Aloosh A (2019). Economic sanctions threaten population health: the case of Iran. Public Health.

[12] Hajebi A, Sharifi V, Abbasinejad M, Asadi A, Jafari N, Ziadlou T (2021). Integrating Mental Health Services into the Primary Health Care System: The Need for Reform in Iran. Iran J Psychiatry.

[13] König H, König HH, Konnopka A (2019). The excess costs of depression: a systematic review and meta-analysis. Epidemiol Psychiatr Sci.

[14] Carta MG, Hardoy MC, Kovess V, Dell’Osso L, Carpiniello B (2003). Could health care costs for depression be decreased if the disorder were correctly diagnosed and treated?. Soc Psychiatry Psychiatr Epidemiol.

[15] Johnston K, Westerfield W, Momin S, Phillippi R, Naidoo A (2009). The direct and indirect costs of employee depression, anxiety, and emotional disorders--an employer case study. J Occup Environ Med.

[16] Luppa M, Heinrich S, Angermeyer MC, König HH, Riedel-Heller SG (2007). Cost-of-illness studies of depression: a systematic review. J Affect Disord.

[17] Berto P, D’Ilario D, Ruffo P, Di Virgilio R, Rizzo F (2000). Depression: cost-of-illness studies in the international literature, a review. J Ment Health Policy Econ.

[18] Ghamari SH, Mohebi F, Abbasi-Kangevari M, Peiman S, Rahimi B, Ahmadi N (2023). Patient experience with chronic obstructive pulmonary disease: a nationally representative demonstration study on quality and cost of healthcare services. Front Public Health.

[19] Abbasi-Kangevari M, Mohebi F, Ghamari SH, Modirian M, Shahbal N, Ahmadi N (2023). Quality and cost of healthcare services in patients with diabetes in Iran: Results of a nationwide short-term longitudinal survey. Front Endocrinol (Lausanne).

[20] Parsaeian M, Mahdavi M, Saadati M, Mehdipour P, Sheidaei A, Khatibzadeh S (2021). Introducing an efficient sampling method for national surveys with limited sample sizes: application to a national study to determine quality and cost of healthcare. BMC Public Health.

[21] Shahraz S, Shahin S, Farzi Y, Modirian M, Shahbal N, Azmin M (2023). Iran Quality of Care in Medicine Program (IQCAMP): Design and Outcomes. Arch Iran Med.

[22] American Psychiatric Association. Diagnostic and Statistical Manual of Mental Disorders, Fifth Edition. American Psychiatric Publishing; 2013.

[23] Samimi Ardestani SM, Davari Ashtiani R, Rezaei Z, Vasegh S, Gudarzi SS. Validation of Persian version of PHQ-9 for diagnosis of major depressive episode in psychiatric wards in Iran. Int J Appl Behav Sci. 2018;5(2).1.

[24] Borges G, Nock MK, Haro Abad JM, Hwang I, Sampson NA, Alonso J (2010). Twelve-month prevalence of and risk factors for suicide attempts in the World Health Organization World Mental Health Surveys. J Clin Psychiatry.

[25] Amin-Esmaeili M, Motevalian A, Rahimi-Movaghar A, Hajebi A, Hefazi M, Radgoodarzi R (2014). The translation and psychometric assessment of the persian version of the sheehan disability scale. Iran J Psychiatry.

[26] Rahimi-Movaghar A, Amin-Esmaeili M, Sharifi V, Hajebi A, Radgoodarzi R, Hefazi M (2014). Iranian mental health survey: design and field proced. Iran J Psychiatry.

[27] Sheehan DV, Nakagome K, Asami Y, Pappadopulos EA, Boucher M (2017). Restoring function in major depressive disorder: A systematic review. J Affect Disord.

[28] Sheehan KH, Sheehan DV (2008). Assessing treatment effects in clinical trials with the discan metric of the Sheehan Disability Scale. Int Clin Psychopharmacol.

[29] Stein DJ, Khoo JP, Picarel-Blanchot F, Olivier V, Van Ameringen M (2021). Efficacy of Agomelatine 25-50 mg for the Treatment of Anxious Symptoms and Functional Impairment in Generalized Anxiety Disorder: A Meta-Analysis of Three Placebo-Controlled Studies. Adv Ther.

[30] National Quality Forum. NQF: Measures, Reports & Tools. Accessed July 13, 2022. Available from: https://www.qualityforum.org/Qps/QpsTool.aspx.

[31] CQAIMH. All eCQMs associated with: Center for Quality Assessment & Improvement in Mental Health (CQAIMH). Accessed July 13, 2022.

[32] Gelenberg AJ, Freeman MP, Markowitz JC (2010). American Psychiatric Association Practice Guideline for the Treatment of Patients With Major Depressive Disorder, Third Edition. Am J Psychiatry.

[33] Jo C (2014). Cost-of-illness studies: concepts, scopes, and methods. Clin Mol Hepatol.

[34] Amin-Esmaeili M, Hefazi M, Radgoodarzi R, Motevalian A, Sharifi V, Hajebi A (2017). Out-of-pocket cost of drug abuse consequences: results from Iranian National Mental Health Survey. East Mediterr Health J.

[35] Gautam S, Jain A, Gautam M, Vahia VN, Grover S (2017). Clinical Practice Guidelines for the management of Depression. Indian J Psychiatry.

[36] Mohamadi E, Takian A, Olyaeemanesh A, Rashidian A, Hassanzadeh A, Razavi M (2020). Health insurance benefit package in Iran: a qualitative policy process analysis. BMC Health Serv Res.

[37] Karimi M (2007). The status of social insurance in the law of the general system of welfare and social security. Soc Secur J.

[38] Doshmangir L, Bazyar M, Rashidian A, Gordeev VS (2021). Iran health insurance system in transition: equity concerns and steps to achieve universal health coverage. Int J Equity Health.

[39] World Bank Group. PPP conversion factor, GDP (LCU per international $)- Iran, Islamic Rep. https://data.worldbank.org/indicator/PA.NUS.PPP?locations=IR. Published 2018. Accessed July 16, 2022.

[40] World Bank Group. GDP per capita, PPP (current international $) - Iran, Islamic Rep. https://data.worldbank.org/indicator/NY.GDP.PCAP.PP.CD?locations=IR. Published 2018. Accessed August 4, 2023.

[41] Keshavarz K, Hedayati A, Rezaei M, Goudarzi Z, Moghimi E, Rezaee M (2022). Economic burden of major depressive disorder: a case study in Southern Iran. BMC Psychiatry.

[42] Greenberg PE, Fournier AA, Sisitsky T, Simes M, Berman R, Koenigsberg SH (2021). The Economic Burden of Adults with Major Depressive Disorder in the United States (2010 and 2018). Pharmacoeconomics.

[43] Tanner JA, Hensel J, Davies PE, Brown LC, Dechairo BM, Mulsant BH (2020). Economic Burden of Depression and Associated Resource Use in Manitoba, Canada. Can J Psychiatry.

[44] Olyaaeemanesh A, Jaafaripooyan E, Abdollahiasl A, Davari M, Mousavi SM, Delpasand M (2021). Pharmaceutical subsidy policy in Iran: a qualitative stakeholder analysis. Health Res Policy Syst.

[45] Statistical Centre of Iran. Iran statistical yearbook 2019-2020: Household expenditure & income. https://www.amar.org.ir/Portals/1/yearbook/1398/21.pdf. Published 2019. Accessed August 4, 2023.

[46] American Psychological Association. APA Clinical Practice Guideline for the Treatment of Depression Across Three Age Cohorts. Washington, DC; 2019. In:2021.

[47] Houle J, Beaulieu MD, Lespérance F, Frasure-Smith N, Lambert J (2010). Inequities in medical follow-up for depression: a population-based study in Montreal. Psychiatr Serv.

[48] Waxmonsky JA, Thomas M, Giese A, Zyzanski S, Dickinson LM, McGinnis GF (2012). Evaluating Depression Care Management in a Community Setting: Main Outcomes for a Medicaid HMO Population with Multiple Medical and Psychiatric Comorbidities. Depress Res Treat.

[49] Busch SH, Leslie D, Rosenheck R (2004). Measuring quality of pharmacotherapy for depression in a national health care system. Med Care.

[50] Akincigil A, Bowblis JR, Levin C, Walkup JT, Jan S, Crystal S (2007). Adherence to antidepressant treatment among privately insured patients diagnosed with depression. Med Care.

[51] Carlo AD, Chan G, Arao RF, Vredevoogd MA, Fortney JC, Powers DM (2021). Assessing the Impact of Different Depression Treatment Success Metrics on Organizational Performance. Psychiatr Serv.

[52] Neslusan C, Chen YW, Sharma M, Voelker J (2023). Unmet need in major depressive disorder and acute suicidal ideation or behavior: findings from a longitudinal electronic health record data analysis. J Med Econ.

[53] Mojtabai R, Amin-Esmaeili M, Spivak S, Olfson M. Remission and Treatment Augmentation of Depression in the United States. J Clin Psychiatry 2021;82(6). doi: 10.4088/JCP.21m13988. 34727421

[54] Cole S, Reims K, Kershner L, McCombs HG, Little K, Ford DE (2012). Improving care for depression: performance measures, outcomes and insights from the Health Disparities Collaboratives. J Health Care Poor Underserved.

[55] McIntyre RS, Lee Y, Mansur RB (2015). Treating to target in major depressive disorder: response to remission to functional recovery. CNS Spectr.

[56] Langlieb AM, Guico-Pabia CJ. Beyond symptomatic improvement:assessing real-world outcomes in patients with major depressive disorder. Prim Care Companion J Clin Psychiatry 2010;12(2). doi: 10.4088/PCC.09r00826bl. PMC2911007 20694113

